# Morphology of Coatings Deposited by Pulsed Electron Deposition Method from Polytetrafluoroethylene-Carbon Composites

**DOI:** 10.3390/molecules30071474

**Published:** 2025-03-26

**Authors:** Agata Niemczyk, Sebastian Fryska, Dariusz Moszyński, Daniel Deacu, Paweł Kochmański, Jolanta Baranowska

**Affiliations:** 1 Department of Materials Technology, Faculty of Mechanical Engineering and Mechatronics, West Pomeranian University of Technology in Szczecin, Piastów Avenue 19, 70-310 Szczecin, Poland; 2 Department of Chemical and Environment Engineering, Faculty of Chemical Technology and Engineering, West Pomeranian University of Technology in Szczecin, Pułaskiego 10, 70-322 Szczecin, Poland

**Keywords:** pulsed electron beam deposition, PTFE/C composites, ablation mechanism, chemical structure

## Abstract

PTFE/C composite targets were ablated using a pulsed electron beam of different energies to evaluate the suitability of this technique for composite coating deposition. Composite materials with two different carbon fillers and their contents (graphite—10 wt.% and coal coke—35 wt.%) were used. A PTFE target was used as reference material. The chemical and physical structure of the coatings was investigated using FTIR, XPS, and XRD. The topography was investigated using optical microscopy, SEM, and AFM. In addition, the contact angle and surface energy of the coatings were evaluated. It was shown that the presence of carbon particles in the polymer matrix decreased the deposition rate but greatly reduced the degradation of PTFE. It is hypothesized that the high content of conductive particles reduces the capability of the pulsed electron beam process to maintain the integrity of the filler particles during the coating deposition process.

## 1. Introduction

In the field of polymer materials, physical vapor deposition (PVD) techniques offer a valuable alternative for coating manufacturing. PVD methods enable the deposition of materials that are difficult to coat, such as polytetrafluoroethylene (PTFE) and many insoluble polymers, which are otherwise challenging and costly to process using conventional techniques. Depending on the specific PVD technique employed, PTFE coatings can exhibit a range of properties, including high crystallinity [1], significant hydrophobicity [2], unique optical characteristics [3], or even rubber-like behavior [4]. However, composite coatings, which combine the advantages of both the matrix and the filler, can be even more attractive, as they offer enhanced mechanical, electrical, or thermal properties. The fabrication of PTFE-based composite coatings, however, presents a significant challenge due to the processing difficulties of PTFE. Consequently, PTFE derivatives such as poly(vinylidene fluoride) (PVDF) and fluorinated ethylene propylene (FEP) are often used, as they offer improved processability. Nevertheless, the production of such coatings still requires multiple processing steps or combined techniques [5,6,7]. Developing a waste-free and solvent-free method for their direct, one-step deposition remains, so far, a challenging goal.

Our previous research demonstrated the feasibility of using the pulsed electron deposition (PED) method for fabricating both PTFE coatings [4,8] and polypropylene-based composite coatings [9]. In the composite coating study, we utilized three polypropylene-based targets, each containing a different type of carbon filler—carbon black, graphene, or carbon nanotubes. The results confirmed that PED is a viable approach for polymer composite deposition, as it preserves the structural integrity of the polymer whilst successfully incorporating functional fillers. Furthermore, the study provided insights into how the type of filler influences coating morphology and structure [9]. These findings highlighted the significant potential of PED for the fabrication of advanced polymer coatings and motivated further investigation into its capabilities for composites with higher filler concentrations.

The deposition of coatings using a target with a higher filler concentration could be an important technological approach. However, until now, no studies have been conducted on the PED of polymer–carbon composite coatings with a high filler content (>10%), where the enhanced thermal and electrical conductivity of the target could play a crucial role. PED is primarily recommended for dielectric materials. When ablating highly electrically and thermally conductive materials, a key challenge is the reduction in the specific energy density at the target, which in turn limits the efficiency of the ablation process [10]. One potential approach to counteract this specific energy density reduction is to apply higher voltages to the electron gun, thereby increasing the beam’s power. However, high power may have a destructive effect on the polymeric matrix material.

Therefore, in this study we have investigated the deposition of PTFE-based composite coatings using the PED method. The target materials contained either 10 wt.% graphite or 35 wt.% carbon coke, and to compensate for the reduction in specific energy density at the target, a high voltage ranging from 12 to 18 kV was applied. The physical and chemical structures of the coatings obtained were characterized in a systematic way. The physical structure and morphology were analyzed using optical microscopy, atomic force microscopy, and X-ray diffraction. The chemical composition was determined via infrared spectroscopy and X-ray photoelectron spectroscopy. The deposition efficiency was evaluated based on coating thickness measurements. Additionally, the coatings were examined by Raman spectroscopy and Scanning Transmission Electron Microscopy combined with Energy-Dispersive Spectroscopy (STEM-EDS).

## 2. Results

Using the PED technique, two series of composite coatings with a polytetrafluoroethylene (PTFE) matrix filled with a carbon filler were produced. The first series, PTFE_GR, was made from a target filled with 10 wt.% of graphite, and the second, PTFE_C, from a target filled with 35 wt.% of carbon coke. As a reference, a series of coatings obtained from PTFE without a filler was also produced. The processes were conducted at constant nitrogen pressure but with various working electron gun voltages. Increasing the electron gun voltage at a pressure of 12 mTorr, led to an increase in the electron beam power. The main process parameters, along with the estimated power and designations of the individual coatings, are listed in Table 1.

**Figure 1:**
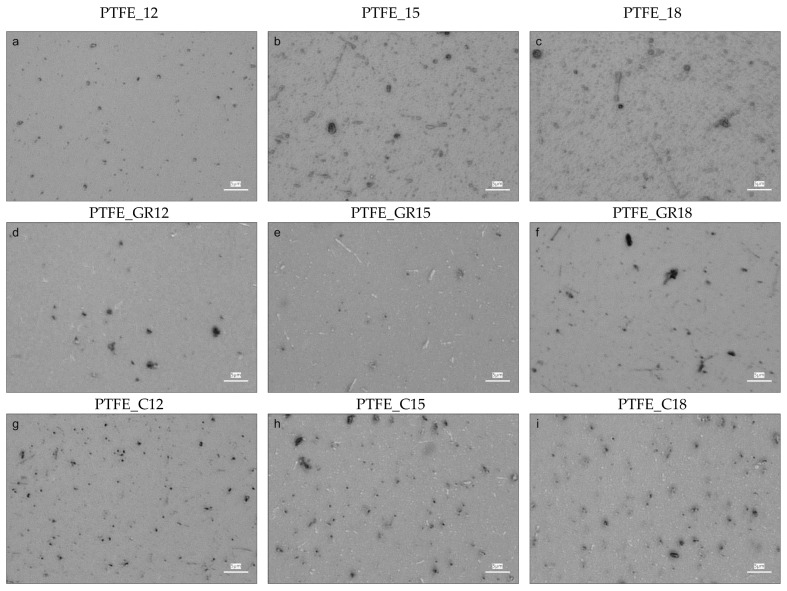
Optical micrographs of PTFE (**a**–**c**), PTFE_GR (**d**–**f**), and PTFE_C (**g**–**i**) coatings obtained at 12, 15, and 18 kV, respectively. The micrographs’ scale bars are equal to 5 µm.

**Figure 2:**
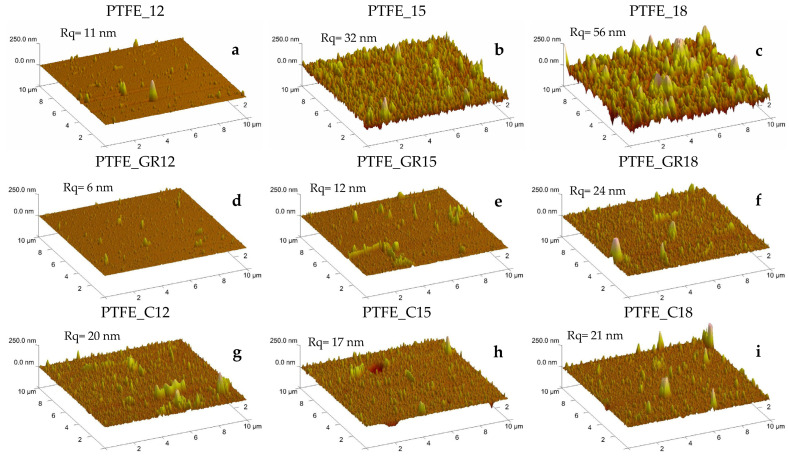
AFM topography scans of PTFE (**a**–**c**), PTFE_GR (**d**–**f**), and PTFE_C (**g**–**i**) coatings obtained at 12, 15, and 18 kV, respectively.

The morphology and physical structure of the coatings obtained were evaluated based on surface imaging using optical and atomic force microscopy (AFM), wettability using contact angle (CA), and crystallinity using X-ray diffraction (XRD). Optical microscopy and AFM images are presented in Figure 1 and Figure 2, respectively. As can be seen in these figures, all the coatings were characterized by the presence of various particles on the surface, but are different in quantity and size depending on the target material (PTFE, PTFE_GR, and PTFE_C) and process conditions.

The images of the PTFE coatings (Figure 1a–c and Figure 2a–c) demonstrate that the surface topography varies with the applied voltage during the PED process, with the lowest voltage PTFE_12 differing the most from the other two PTFE coatings. A higher voltage leads to an increased roughness (Rq) of the PTFE coatings, ranging from Rq = 11 nm for PTFE_12 to Rq = 56 nm for PTFE_18. A similar trend was observed by Gupta et al. [11] for PTFE coatings produced at 15 mTorr of nitrogen with an increasing voltage. This effect can be attributed to the fact that at a pressure of 12 mTorr (and 15 mTorr), increasing the voltage from 12 to 18 kV results in higher electron beam power, which alters the ablation. According to our hypothesis on the production of PTFE coatings via the PED method [12], the physical and chemical structure of the coatings depends on the electron beam power used. Different beam power levels lead to variations in the ablation mechanisms, affecting target penetration depth and interactions with PTFE macromolecules. During ablation, these interactions generate diverse atomic groups with varying capacities for macromolecular reformation in the growing coating [12].

A change in topography was particularly pronounced in the coatings produced from unfilled PTFE, where the roughness increased as the applied voltage increased. A similar trend was observed for PTFE_GR series, but the roughness was smaller (Figure 2d–f). For the PTFE_C coatings, no clear correlation with voltage was observed.

In addition to topographical differences, the coatings also varied in thickness. PTFE coatings without filler showed the greatest thickness, ranging from 170 to 700 nm, while PTFE_GR coatings ranged from 80 to 120 nm, and PTFE_C coatings from 50 to 90 nm (Table 2). Stated in a simplified way, increasing electron beam power should enhance ablation and result in thicker coatings. Such a trend is evident in the PTFE and PTFE_C series: coatings produced at 12 kV were the thinnest, while those produced at 18 kV were the thickest (Table 2). Nevertheless, in terms of the amount of ablated and subsequently deposited material per electron beam pulse (deposition efficiency), there is a significant difference between unfilled PTFE and both of the PTFE composites. The presence of a thermally conductive carbon filler drastically reduces the ablation rate, decreasing the amount of material removed per pulse by nearly an order of magnitude.

**Figure 3:**
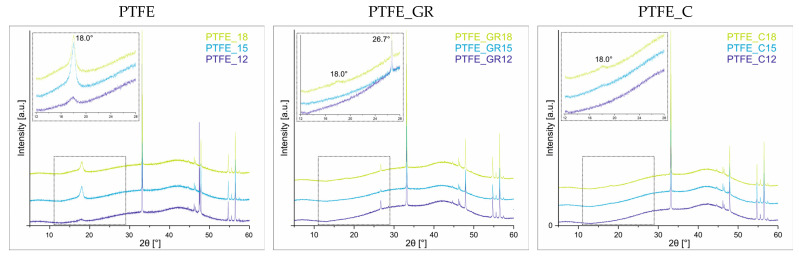
XRD of PTFE, PTFE_GR, and PTFE_C series.

The crystallinity of the coatings was evaluated using XRD analysis. The diffraction patterns for all three series of coatings are presented in Figure 3. Only in the PTFE series do all three coatings exhibit a characteristic diffraction peak at 2θ = 18°. Notably, the intensity of this peak is higher for PTFE_15 and PTFE_18, suggesting that the observed changes in surface topography (Figure 2a–c) may be partially attributed to increased crystallinity and the formation of more ordered crystalline structures.

In contrast, for the composite coatings, the diffraction peak at 2θ = 18° appears only in those coatings produced at the highest voltage (PTFE_GR18 and PTFE_C18), indicating that the composite coatings exhibit a predominantly amorphous nature. Naturally, the lower thickness of the composite coatings also contributes to the reduced intensity of this peak. For the PTFE_GR series, an additional diffraction peak at 2θ = 26.7°, corresponding to the graphite (002) reflection [13] is observed, confirming the presence of graphite in the coatings. All other diffraction peaks (2θ = 33.1, 46.3, 46.5, 49.9, 54.8, 55.6, 56.5, 57.5°) originate from the Si substrate. The diffraction pattern of the Si substrate is shown in the Supplementary Materials in Figure S1.

**Figure 4:**
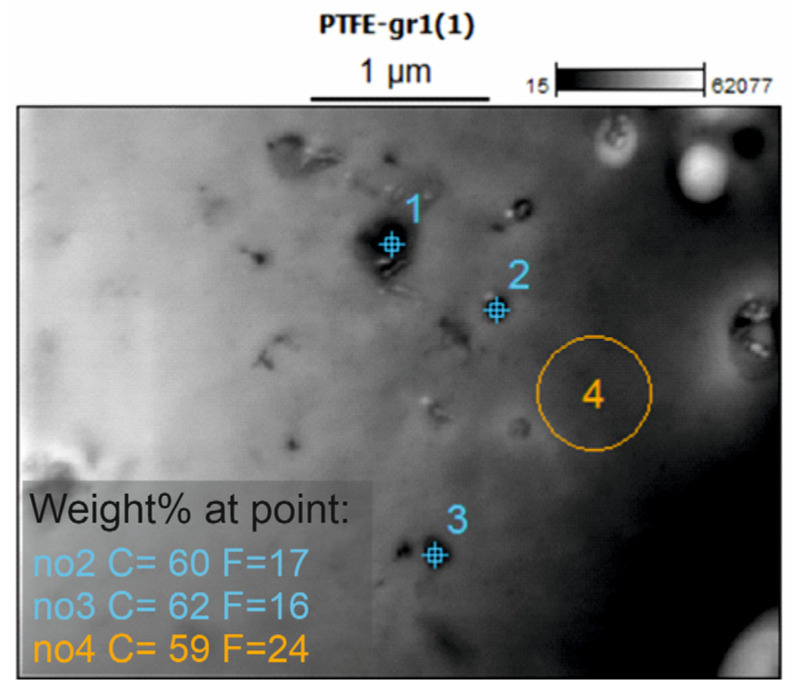
STEM micrograph of PTFE_GR1 with chemical microanalysis results (EDS) at selected points.

In an attempt to confirm and visualize the presence of the carbon filler in the composite coatings, the coatings were subjected to STEM EDS analysis. Unfortunately, the composite coatings did not demonstrate adequate mechanical strength and integrity to enable proper examination. Only the PTFE_GR12 coating was successfully measured, and the result is presented in Figure 4. STEM micrographs of PTFE_GR15 and PTFE_GR18 are presented in Figure S2 in the Supplementary Materials. Signal intensity vs. the energy of the three measured points of PTFE_GR12 is also shown in Figure S2. As can be seen in Figure 4, particles of various sizes can be observed in the PTFE_GR12 coating. The elemental composition of these particles revealed that the particle region has a lower fluorine content than the plain region without particles, indicating that the particles observed are most likely the carbon filler. Nevertheless, this is not completely conclusive evidence, and the coatings were therefore subjected to Raman spectroscopy in the region 1200–1700 cm^−1^, which is a typical range in which peaks from carbon should be present. Unfortunately, even though different laser wavelengths were used as well as varying other measurement parameters, none of the recorded spectra for the composite series showed the expected peaks. This may be attributed to the low thickness of the coatings combined with the low amount of carbon filler. Nevertheless, XRD analysis confirms that graphite is present in the material structure of PTFE_GR coatings.

To investigate the chemical structure of the coatings obtained, infrared spectroscopy was performed on both the coatings and the target materials from which they were produced. Figure S3 (Supplementary Materials) presents the spectra of the starting materials, i.e., the PTFE, PTFE_GR, and PTFE_C targets. Due to the large amount of carbon filler in the PTFE_C target (which absorbs the IR beam), its spectrum exhibits much lower intensity in addition to significant noise. However, all three targets have practically identical IR spectra, confirming that the chemical structure of PTFE remains unchanged before the PED process.

**Figure 5:**
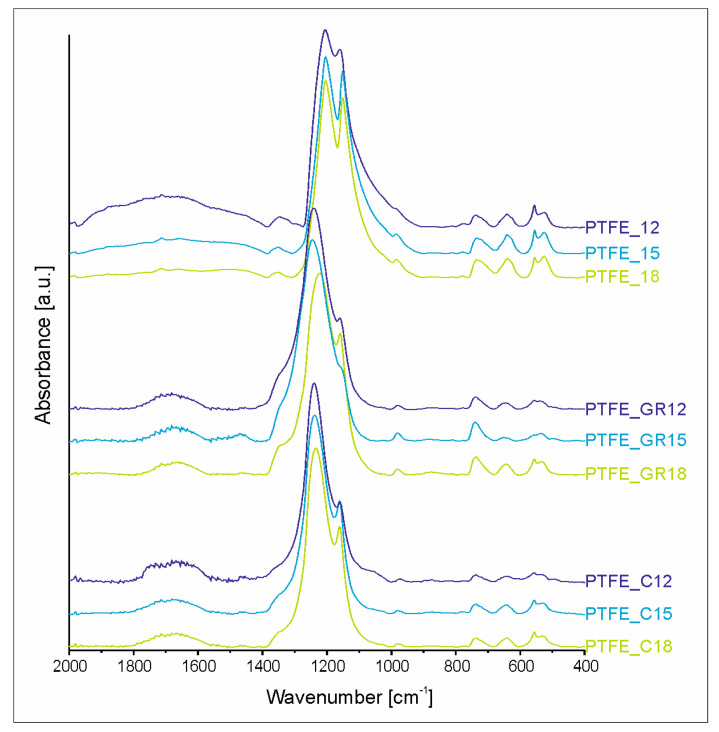
FTIR spectra of the PTFE, PTFE_GR, and PTFE_C series coatings.

The spectra of all coatings (Figure 5) have the typical IR spectral profile of PTFE material, with the most intense absorption bands in the range of 1250–1100 cm^−1^. However, the high voltage applied during the PED process induces structural changes, which differ depending on whether the target material contains a carbon-type filler or not.

To evaluate the spectral changes quantitatively the region 2000–400 cm^−1^ was deconvoluted. Due to the many new bands present in spectra as well as their broadness, individual bands and regions representing similar changes in the chemical structure are distinguished. The results of deconvolution, i.e., the percentage of the area of bands or specified regions, are presented in Table 3. Except for the band at 1878 cm^−1^, all the same bands are contained within the spectra of the coatings, indicating that the chemical structure of the coatings is built up of the same types of bonds and functional groups. However, their amount in the structure differs significantly between coatings from the PTFE and composite targets. The first difference lies in the presence of the band 1878 cm^−1^ in the PTFE coating without carbon-based filler, suggesting that the acid fluoride group (COF) can be present. The literature indicates that this group can be formed even after the irradiation process is completed, provided that radicals are present in the material [14]. Another difference is in the amount of unsaturated C=C bonds evidenced by absorption bands in region 1718–1460 cm^−1^. Bands at 1730, 1717, and 1671 cm^−1^ correspond to the –CF=CF_2_, –CF=CF–, and –CF=C< bonds [15]. The presence of these spectral features suggests partial degradation of the PTFE chain, which is typically observed in PTFE exposed to highly energetic radiation [14,15]. The amount of CF_3_ groups in the PTFE coatings, represented by band from 1390 to 1300 cm^−1^ region, is quite low in contrast to the composite coatings, suggesting low chain branching in the PTFE coatings. A further indication that the PTFE coating structure has a lower degree of branching or cross-linking than the composite coating structures is the ratio of the band characteristic for C-C bonds (at 1237 cm^−1^) to the band of CF_2_ groups (at 1205 and 1056 cm^−1^). For PTFE from the target, this ratio is 4.3, compared to between 2.4 and 4.0 for the PTFE coatings. This low branching/crosslinking degree distinguishes the PTFE coatings prepared for this work from those previously reported, which were characterized by rubber-like properties [12]. We attribute this difference in chemical structure to the influence of the electron beam power, which was higher in the present study. In general, increasing the electron beam power (or other types of high energetic beam/radiation exposure) is associated with stronger PTFE chain degradation and the formation of a greater number of unsaturated bonds. The consequence is the shortening of the PTFE chains, which simultaneously leads to increased crystallinity due to the increased mobility of shorter chains [16,17].

The IR spectra of the PTFE_GR and PTFE_C series are quite similar but differ from the spectra of the starting target materials and the PTFE coatings without fillers. The main difference is that the most intense band in the region 1250–1100 cm^−1^ is the band at 1230 cm^−1^ originating from C-C bonds. This means that the branching and cross-linking of PTFE in the composite coatings is much greater. However, such a large amount of C-C bonds also suggests that partial degradation of the filler could have occurred; the atomic splitting of the carbon fillers and their partial incorporation into the polymer chain. This would explain the problems encountered with obtaining a Raman spectrum. The increased intensity of bands in the 1390–1300 cm^−1^ region is consistent with the occurrence of chain branching. This suggests that the chemical structure of these coatings may be similar to those exhibiting rubber-like properties [9]. This is in line with the XRD results, which indicate higher amorphousness of these samples. The significantly lower number of double bonds indicates significantly less PTFE chain degradation.

The effect of the process conditions as well as the presence of the carbon filler in the target is also demonstrated by the surface composition of the materials as determined by XPS studies. The X-ray photoelectron survey spectra of the composite coatings are shown in Figure S4 in the Supplementary Materials. In the case of the PTFE_GR series of coatings, an increase in beam power results in an elevated concentration of surface oxygen. This means that after the process, more free radicals remain on the surface, which can undergo oxidation reactions. It is interesting to note that in the case of the PTFE_C series, the voltage, and thus the beam power, has very little effect on the elemental composition of these coatings. The difference in the percentage of oxygen between the PTFE_C coatings is 0.8% (compared to 2.1% for PTFE_GR) and regarding fluorine 1.1% compared to 4.6%. Interestingly, although the coatings have a significantly higher proportion of C-C bonds in the structure (Table 4), the surface composition of all the composite coatings is very close to the values of the reference material.

The morphology of the coatings can directly impact functional surface properties, such as wettability. To analyze the influence of the PED process parameters and the presence of the filler on the functional surface properties, contact angle measurements were conducted. One of the main functional properties of PTFE surfaces is their high hydrophobicity, which can enhance corrosion resistance, reduce friction between contacting surfaces, and improve biocompatibility [18,19,20].

**Figure 6:**
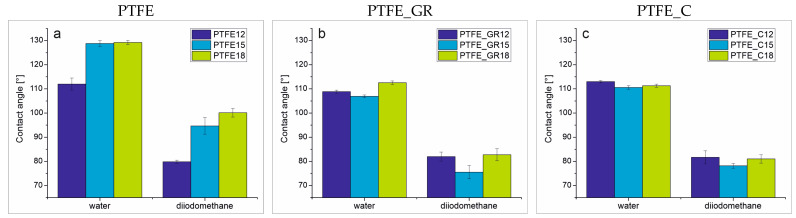
Contact angle of PTFE, PTFE_GR, and PTFE_C series coatings.

For the wettability study, contact angles were measured using water and diiodomethane as test liquids. The measured contact angle values, along with the standard deviation, are presented in Figure 6. All of the composite coatings exhibited hydrophobic surfaces, with similar contact angle values within each series for both test liquids. Specifically, the contact angle was approximately 110° for water and 80° for diiodomethane. In contrast, unfilled PTFE coatings exhibited significantly higher contact angles, reaching approximately 130° for water and 100° for diiodomethane depending on the applied voltage. This higher contact angle is likely a result of the increased roughness and the relatively regular topographical patterns observed in these coatings (Figure 2b,c).

For further understanding of the surface properties, the Owens–Wendt method was used to calculate the surface energy of the coatings, including their dispersive (γ_D_) and polar (γ_P_) components. These values are summarized in Table 5. For reference, the surface energy of bulk PTFE is 18.7 mN/m, with γ_D_ = 17.3 ± 1.0 mN/m and γ_P_ = 1.4 ± 0.6 mN/m [21]. The values obtained for all coatings, except for PTFE_15 and PTFE_18, are relatively similar. Notably, the polar component of the surface energy is nearly zero. The different results for the PTFE15 and PTFE18 are likely due to the higher roughness of these coatings and hence decreased contact between the drop and the surface; however, the presence of carbon filler may also play a role [22]. The reduced surface energy and dispersive component values (a high contact angle for water and diiodomethane) observed for PTFE_15 and PTFE_18 indicate that these coatings show properties towards omniphobic coatings.

## 3. Discussion

The introduction of carbon filler into the PTFE matrix resulted in a significant reduction in the ablation rate. This may be related to a significant change in the volume of the material within which the electron beam is absorbed. In materials exposed to a high-energy electron beam, its absorption occurs at a depth D + D_T_, where D is the electron absorption range [μm] and D_T_ is the thermal diffusion length [μm]. The rate of temperature increase (dT/dt), which is important from the viewpoint of the ablation rate, is therefore directly proportional to the density of the absorbed energy in the volume of the material [23]:

$$\frac{dT}{dt}\sim \frac{Q}{D+D_{T}}$$

where Q is the power density [Wcm^−2^].

The electron absorption range is strongly dependent on the electron energy ε [keV] [10] and can be expressed as:

$$D=4.57\times 10^{-2}\cdot \varepsilon^{1.74}\cdot \frac{1}{\rho }[\mu m]$$

where ε is the electron energy [keV] and ρ is the material density [gcm^−3^].

The thermal diffusion length can be expressed as:

$$D_{T}=2\cdot (\alpha \cdot \tau {)}^{1/2}$$

where α is the thermal diffusivity [m^2^·s^−1^] and τ is the pulse time [s].

For dielectrics, D is usually much larger than D_T_. In the case of PTFE ablation, D increased from 1.57 to 3.17 μm depending on the applied electron beam energy, while D_T_ was 0.16 μm. The increase in electron energy (from 12 to 18 keV) was accompanied by a significant increase in beam power (from 0.25 to 2.0 MW, Table 1), which despite the increased absorption volume ensured threshold conditions for the ablation process. As a result, the deposition efficiency increased significantly (Table 2).

The introduction of carbon fillers to the PTFE matrix increases D (because of a slightly lower filler density compared to the polymer matrix) and increases D_T_, due to an increase in thermal diffusivity of the composite compared to unfilled PTFE (Table 6). The calculation methodology for the values presented in Table 6 is included in the supplementary materials.

As a result, the average value of the specific power density decreases, which could be decisive from the point of view of the deposition efficiency of coatings with filler (Table 2). Nevertheless, comparing the thicknesses of coatings (Table 2) with the specific power density for the tested materials (Table 6), it is difficult to find a good correlation. For example, the similar specific power density for the PTFE_12, PTFE_GR18, and PTFE_C15 coatings was not reflected in the thickness, which is clearly different for these materials (170, 90, and 50 nm, respectively).

Moreover, these coatings differ significantly in the chemical structure of the PTFE chains. The PTFE structure of coatings produced from unfilled targets is characterized by a significant number of unsaturated bonds, which in the case of PTFE are a sign of macromolecule degradation. On the contrary, composite coatings have a much smaller number of unsaturated bonds, with a branched/cross-linked structure. This indicates a more destructive nature of the electron beam interaction with the ablated material without filler, even though the calculations indicate that the specific power density has a similar level as that of polymer material with filler.

The observed ambiguities may be the effect of the increased electrical conductivity of the composites investigated. For the PTFE_GR composite, the graphite content was within the percolation region [24,25], while for PTFE_C, the carbon content places this composite within the conductivity region [26], which was reflected in the measured values of the electrical conductivity (Table 6). Regarding the PTFE_C samples, the studies did not confirm the unequivocal presence of filler particles in the coatings. Moreover, based on the FTIR results, the possibility of incorporating carbon from the filler into the polymer structure cannot be ruled out. This could suggest that in the case of a composite with conductive properties, the ablation mechanism changes. However, further studies are required to confirm this hypothesis.

Our previous studies have shown that by using PED for ablation of a polymer–carbon filler composite [9], it is possible to transfer the composite material with good preservation of both the chemical structure and filler particles. In those studies, polypropylene was used, which has a much lower density than PTFE (PP density = 0.9 g/cm^3^, PTFE density = 2.2 g/cm^3^) and a lower volume fraction of carbon filler was used in the matrix. Based on the conducted studies, it was suggested that in the case of polymer–carbon composites, the thermal conductivity of carbon facilitates partial decomposition of the polymer surrounding the filler particle and the subsequent formation of gaseous products is the basis for the spallation mechanism for such large particles. However, the current studies with a much higher filler content in the polymer matrix suggest the following:

1. In the case of performing PED processes with composite materials, the amount of filler may play a significant role and affect the ablation process and thus the characteristics of the coatings produced.
2. A change in electrical conductivity (even at a level not disqualifying the material from being a dielectric) may affect the ablation process and should perhaps be included in models describing ablation and ablation efficiency.

## 4. Materials and Methods

PTFE composite coatings were deposited using the PED system (NEOCERA, Inc., Beltsville, MD, USA). The coatings were deposited on Si substrates at a nitrogen pressure of 12 mTorr, at room temperature. The electron source operated at 12, 15, and 18 kV, with a repetition rate of 5 Hz and 10,000 pulses (preceded by 1000 pre-ablation pulses). The distance between the target and substrate was set at 70 mm, and the distance between the ceramic end of the electron gun and the PTFE target at 18 mm. The targets were 50 mm diameter disks as follows: bulk PTFE with a purity of 99% (^®^, P.H.U. SZCZEL-PLAST S.C., Mikołajów, Poland), PTFE with 10 wt.% of graphite and PTFE with 35 wt.% of carbon coke (Polyfluor, Breda, The Netherlands).

The PTFE structure was analyzed using ATR-FTIR spectroscopy (Tensor, Bruker, Billerica, MA, USA). Each spectrum consisted of 132 scans at a resolution of 4 cm^−1^. Spectra were baseline-corrected and collected in the wave number range 4000–400 cm^−1^. To quantify changes in the individual band intensities to define bonds in the material, deconvolution of the 2000–400 cm^−1^ region was performed using MagicPlot software2.9.3. Gaussian peaks were fitted with a maximum error estimation of less than 5%, and correlation coefficients exceeding 0.995 were achieved during the fitting process. The X-ray photoelectron spectra were obtained using Mg Kα (hν = 1253.7 eV) radiation using a Prevac (Rogów, Poland) system equipped with a Scienta SES 2002 electron energy analyzer operating at constant transmission energy (Ep = 50 eV). The samples were attached to a stainless-steel sample holder. Charging effects were corrected by using the XPS C 1s peak (the binding energy was set to 285.0 eV). Data processing involved background subtraction by means of an “S-type” integral profile and a curve-fitting procedure based on a least-squares method (software CasaXPS 2.3.16). The phase composition of the PTFE coatings was investigated by X-ray diffraction (XRD) using an X’PERT PANalytical diffractometer with CuKα radiation. Bragg–Brentano geometry was employed with a scan step of 0.008° and time per step of 400 s within a 2θ angle range of 5–60°. The layers were examined using light optical microscopy using a VHX-7000 digital microscope (Keyence, Itasca, IL, USA) and scanning electron microscopy using an FE-SEM SU-70 (Field Emission Scanning Electron Microscopy) microscope (Hitachi, Naka, Japan) equipped with EDS. Ar milling was used to obtain a cross-section of the coatings. The milling was carried out for 50 min with an acceleration voltage of 6.0 kV and a gas pressure of 0.15 cm^3^/min. The topography of the layers was examined by atomic force microscopy using a Veeco NanoScope Iva (Veeco, Dornach Munich, Germany) on an area of 10 × 10 µm in contact mode. Static contact angle measurements of the coatings were performed using a Krüss DSA 100 Drop Shape Analyzer goniometer equipped with a camera and recording system. Measurements were performed using 2 μL drops of water and diiodomethane. For each liquid, 5 drops were deposited. The surface energy was calculated using the Owens–Wendt method. Raman spectroscopy (inVia Reflex Renishaw spectrometer, Gloucestershire, UK) was used to characterize the composite coatings. Electrical conductivity measurements were performed on target materials with dimensions of 10 × 10 × 5 mm, which were clamped between copper plate connectors and polarized with a DC voltage ranging from 0 to 200 V. The measurements were conducted using a SUPER MEGOHM METER SM7420 (Hioki Corporation, Nagano, Japan).

## Figures and Tables

**Table 1:** The target material, voltage and beam power used, and coating designation.

Target	Voltage	Beam Power ^1^	Designation
PTFE	12 kV	0.25 MW	PTFE_12
	15 kV	0.86 MW	PTFE_15
	18 kV	2.0 MW	PTFE_18
PTFE + graphite 10 wt.%	12 kV	0.25 MW	PTFE_GR12
	15 kV	0.86 MW	PTFE_GR15
	18 kV	2.0 MW	PTFE_GR18
PTFE + carbon coke 35 wt.%	12 kV	0.25 MW	PTFE_C12
	15 kV	0.86 MW	PTFE_C15
	18 kV	2.00 MW	PTFE_C18

^1^ electron beam power was estimated based on data provided by the manufacturer of the electron gun.

**Table 2:** Thickness and the deposition efficiency of the coatings.

Coating	PTFE_12	PTFE_15	PTFE_18	PTFE_GR12	PTFE_GR15	PTFE_GR18	PTFE_C12	PTFE_C15	PTFE_C18
Thickness [nm]	170 ± 20	500 ± 30	700 ± 120	120 ± 10	80 ± 10	90 ± 10	60 ± 10	50 ± 10	90 ± 10
deposition efficiency [Å/pulse]	0.17	0.50	0.70	0.12	0.08	0.09	0.06	0.05	0.09

**Table 3:** The percentage of the area of individual bands or bands regions for the PTFE, PTFE_GR, and PTFE_C series, calculated from the IR-region deconvolution.

	Percentage of the Individual Bands’ Area Calculated from the IR-Region Deconvolution.								
Wavenumber or Wavenumber Range [cm^−1^]	PTFE_12	PTFE_15	PTFE_18	PTFE_GR12	PTFE_GR15	PTFE_GR18	PTFE_C12	PTFE_C15	PTFE_C18
1878	4.1	1.9	1.3	-	-	-	-	-	-
1718–1460	22.9	13.9	9.1	7.5	7.9	5.6	13.8	8.4	8.2
1390–1300	1.2	0.7	0.6	17.7	14.7	10.5	10.5	8.8	7.5
1244–1233	11.3	9.0	9.3	42.1	48.3	38.4	42.2	47.2	43.2
1205	14.3	19.0	20.0	8.7	7.2	11.4	8.1	7.9	10.8
1181	7.1	7.5	9.4	4.8	4.7	6.2	4.5	6.1	5.1
1156	13.1	16.3	16.7	10.2	8.6	15.1	9.3	11.9	14.1
1126	5.9	6.6	7.8	2.4	2.1	3.4	1.8	1.8	2.2
1095–1040	11.0	12.7	12.1	1.6	0.5	2.1	4.3	2.4	2.8
1015–900	3.8	4.4	4.3	0.3	0.8	0.4	0.3	0.2	0.3
880–700	1.6	2.5	3.3	2.1	3.1	2.9	1.8	1.8	1.7
650–440	3.7	5.6	6.1	2.7	2.3	3.9	3.6	3.7	4.1
CF_2_/C–C	2.4	3.9	4.0	0.4	0.4	0.6	0.4	0.3	0.7

**Table 4:** The surface elemental composition of coatings expressed in at.% calculated based on XPS results.

Element	PTFE Target	PTFE_GR12	PTFE_GR15	PTFE_GR18	PTFE_C12	PTFE_C15	PTFE_C18
C	32.2	35.7	37.9	38.3	38.9	37.7	37.9
F	67.8	63.2	60.2	58.6	59	60.1	59.2
O	0	1	1.9	3.1	2.1	2.2	2.9

**Table 5:** Surface energy and dispersive and polar component values of PTFE, PTFE_GR, and PTFE_C coating series.

Coating	Total SE [mN/m]	Dispersive Component [mN/m]	Polar Component [mN/m]
PTFE_12	17.6 ± 0.4	17.6 ± 0.3	0.0
PTFE_15	11.0 ± 1.5	10.8 ± 1.4	0.3 ± 0.2
PTFE_18	8.7 ± 0.7	8.6 ± 0.6	0.1 ± 0.1
PTFE_GR12	16.7 ± 0.8	16.5 ± 0.9	0.3 ± 0.1
PTFE_GR15	19.7 ± 1.3	19.8 ± 1.6	0.2 ± 0.1
PTFE_GR18	16.1 ± 1.2	16.1 ± 1.2	0.1 ± 0.0
PTFE_C12	16.7 ± 1.4	16.6 ± 1.4	0.0
PTFE_C15	18.4 ± 0.5	18.4 ± 0.5	0.0
PTFE_C18	17.0 ± 0.8	17.0 ± 0.9	0.0

**Table 6:** The D and D_T_ ranges, the resulting specific power densities calculated for target materials depending on the electron energy applied during the deposition processes as well as electrical conductivity.

	PTFE			PTFE_GR			PTFE_C		
Electron energy [keV]	12	15	18	12	15	18	12	15	18
D [μm]	1.57	2.31	3.17	1.59	2.35	3.22	1.65	2.44	3.34
D_T_ [μm]	0.16			0.21			0.20		
Specific power density [kW/μm^3^] *	11.6	14.2	12.9	10.2	12.9	11.8	9.3	11.7	10.7
Electrical conductivity [S/cm]	~10^−18^			2 × 10^−11^			0.027		

* calculated assuming a hemispherical shape of the absorption region with radius D + D_T_.

## Data Availability

The data presented in this study are available on request from the corresponding author due to the fact that the data are part of ongoing research and future project research. As such, controlled access to the data ensures that they are not prematurely disclosed or misinterpreted before additional analyses or publications are completed. We are committed to transparency and scientific rigor and welcome inquiries regarding the data. Please contact the corresponding author for further information or access to the data.

## Supplementary Materials

The following supporting information can be downloaded at: https://www.mdpi.com/article/10.3390/molecules30071474/s1, Figure S1. The diffraction pattern of the Si substrate, Figure S2. Micrographs from the EDS measurement of a—PTFE_GR15 and b—PTFE_GR18, Figure S3. FTIR spectra of the PTFE, PTFE_GR, and PTFE_C target materials. Calculations of the thermal parameters of the tested composites. Table S1. Thermal conductivity, specific heat capacity, and density [26,27,28,29,30,31,32].
