# Supplementary material for: Morphology of Coatings Deposited by Pulsed Electron Deposition Method from Polytetrafluoroethylene-Carbon Composites
Source: Molecules. 2025 Mar 26;30(7):1474. doi: 10.3390/molecules30071474 (PMC11990844; doi:10.3390/molecules30071474)
Supplement: Supplementary file 1 [file molecules-30-01474-s001.zip › molecules-3511833-supplementary.pdf]

Supplementary Materials

Morphology of coatings deposited by Pulsed Electron Deposition method from Polytetrafluoroethylene–carbon composites

Agata Niemczyk [1,*], Sebastian Fryska [1], Dariusz Moszyński [2], Daniel Deacu [1], Paweł Kochmański [1], and Jolanta Baranowska [1,*]

[1] Department of Materials Technology, Faculty of Mechanical Engineering and Mechatronics, West Pomeranian University of Technology in Szczecin, Piastów Avenue 19, 70-310 Szczecin, Poland

[2] Department of Chemical and Environment Engineering, Faculty of Chemical Technology and Engineering, West Pomeranian University of Technology in Szczecin, Pułaskiego 10, 70-322 Szczecin, Poland

* Correspondence: aniemczyk@zut.edu.pl (A.N.); baranops@zut.edu.pl (J.B.)

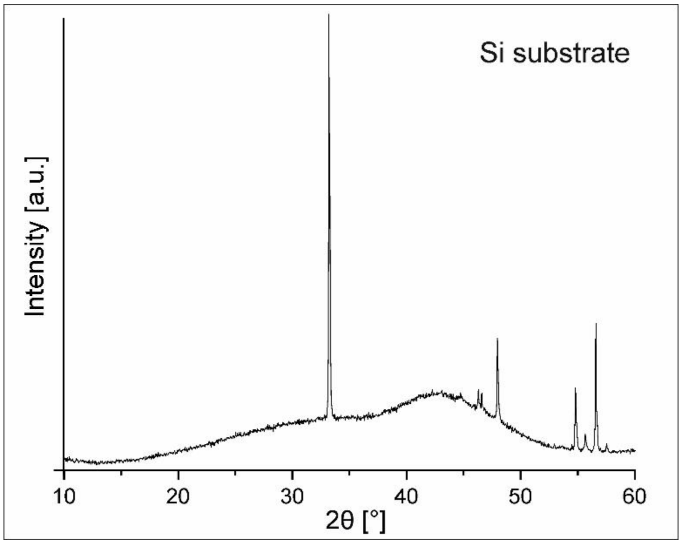

Figure S1. The diffraction pattern of the Si substrate.

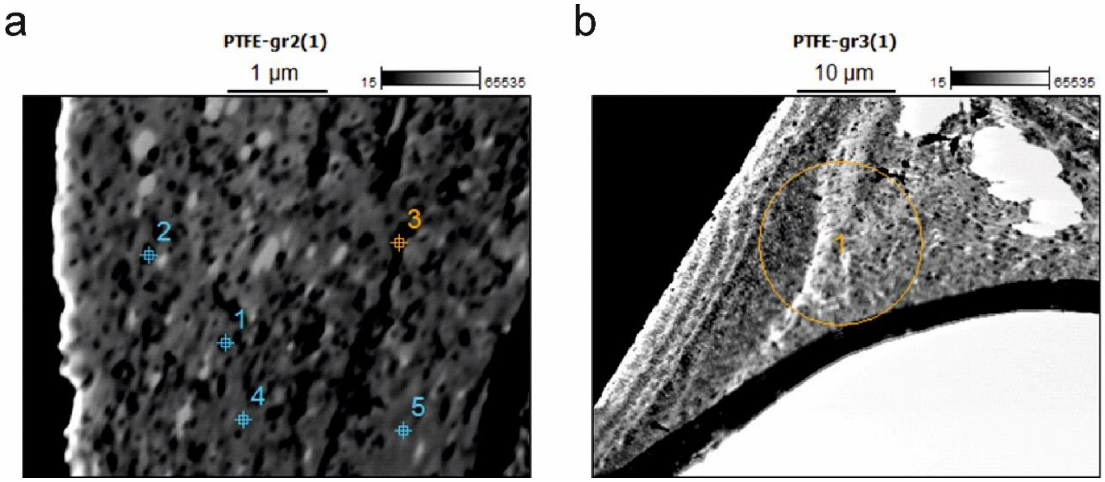

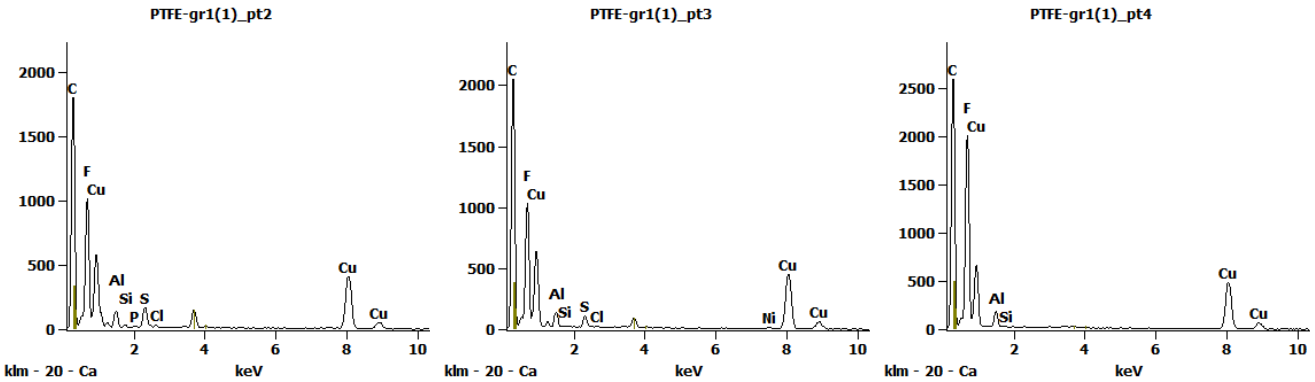

Figure S2. Micrographs from EDS measurement of (a) PTFE_GR15 and (b) PTFE_GR1 and the signal intensity vs. the energy of the three measured points of PTFE_GR12.

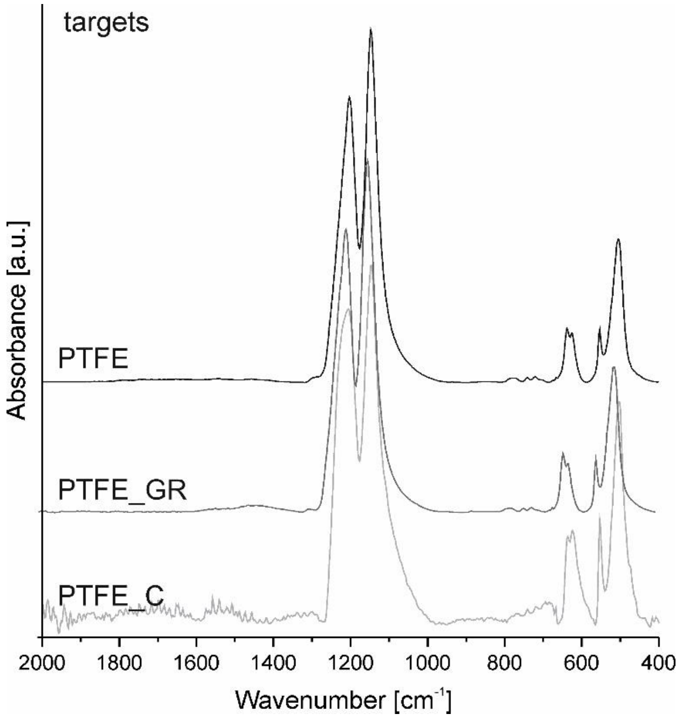

Figure S3. FTIR spectra of the PTFE, PTFE_GR, and PTFE_C target materials.

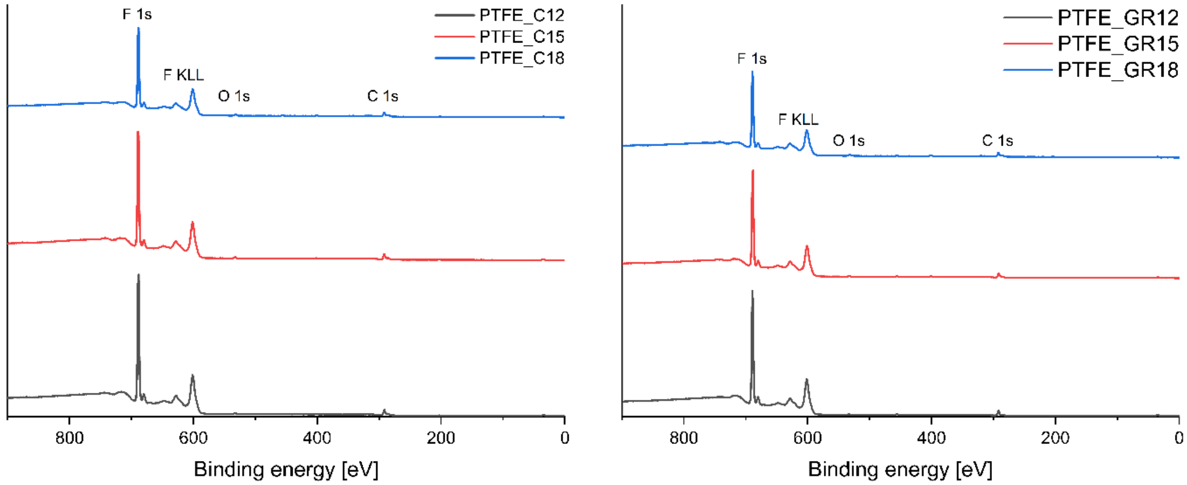

Figure S4. X-ray photoelectron survey spectra of selected materials.

Calculations of thermal parameters for the tested composites.

The table presents thermal parameters of the materials that make up the tested composites, which were used in the calculations.
The calculation of the heat capacity of the composites:

$$c_p^{comp} = f_v^{fil} \cdot c_p^{fil} + (1 - f_v^{fil}) \cdot c_p^{PTFE}$$

Where: C_p is the thermal capacity [J/g*K] of the composite, filler, and PTFE; and f_v^{fil} is the filler volume fraction 0,11 and 0,38, respectively, for graphite and carbon.
The biggest challenge is to calculate the thermal conductivity *K* for the composite material. There are a number of models describing the methods of calculating this value for polymer composites [S2]. For the purposes of these calculations, the model proposed in [S3] was adopted, as it showed good agreement for a wide range of carbon filler contents:

$$k_{comp} = k_{PTFE} \cdot \frac{2 \cdot k_{PTFE} + k_{fil} + 2 \cdot f_v^{fil}\left(k_{fil} - k_{PTFE}\right)}{2 \cdot k_{PTFE} + k_{fil} - 2 \cdot f_v^{fil}\left(k_{fil} - k_{PTFE}\right)}$$

where: k is the thermal conductivity [W/mK] for the composites (k_{comp}), PTFE (k_{PTFE}), and the filler (k_{fil}).

Table S1. Thermal conductivity, specific heat capacity, and density

	PTFE	Gr	CC	PTFE+Gr	PTFE+C
Thermal conductivity [W/mK]	0.210 [S3]	197[S3]	0.31 [S4]	0.333	0.259
Specific heat capacity [J/gK]	1.5 [S6]	0.71 [S1]	0.76 [S5]	1.41	1.22
Density [kg/m^3]	2.2E+6 [S7]	1.9E+6 [S1]	1.9E+6 [S4]	2.17E+6	2.08E+6

The weight fractions for graphite and carbon were f_w^{Gr} =0.1 and f_w^{C} = 0.35, respectively.

The volumetric fractions for graphite and carbon were f_v^{Gr} =0.11 and f_v^{C} = 0.38, respectively.

Reference

1. McEligot, D.M.; Swank, W.D.; Cottle, D.L.; Valentin, F.I. Thermal Properties of G-348 Graphite, OSTI.GOV. **2016**. https://doi.org/10.2172/1330693.
2. Pietrak, K.; Wisniewski, T.S.; A review of models for effective thermal conductivity of composite materials. J. Power Technol.**2015**, *95*, 14–24.
3. Jin, Z.; Chen, X.; Wang, Y.; Wang, D. Thermal conductivity of PTFE composites filled with graphite particles and carbon fibers. Comput. Mater. Sci. **2015**, *102*, 45–50.
4. Soto-Rivero, M.F.; Quintero-Garzón, A.C.; Peña-Rodríguez, G.; Miranda-Molina, L.A.; Sepulveda-Solano, G. Effective thermal properties and proximate analysis of coke-coal fines mixtures. J. Phys.: Conf. Ser. 2021, 2046. https://doi.org/10.1088/1742-6596/2046/1/012045.
5. Ordabaeva, A.T.; Muldakhmetov, Z.M.; Kim, S.V.; Kasenova, S.B.; Sagintaeva, Z.I.; Gazaliev, A.M. Electrophysical Properties and Heat Capacity of Activated Carbon Obtained from Coke Fines. Molecules. **2023**, *28*, 18, doi.org/10.3390/molecules28186545.
6. Available online: https://www.matweb.com/search/datasheet_print.aspx?matguid=4e0b2e88eeba4aaeb18e8820f1444cdb (accessed on 1 March 2025).
7. Blumm, J.; Lindemann, A.; Meyer, M.; Strasser, C. Characterization of PTFE Using Advanced Thermal Analysis Technique. Int. J. Thermophys. **2010**, *31*, 1919–1927. https://doi.org/10.1007/s10765-008-0512-z.
